# Diagnostic accuracy of eHealth literacy measurement tools in older adults: a systematic review

**DOI:** 10.1186/s12877-023-03899-x

**Published:** 2023-03-29

**Authors:** Yu Qing Huang, Laura Liu, Zahra Goodarzi, Jennifer A. Watt

**Affiliations:** 1 Division of Geriatric Medicine, Department of Medicine, University of Toronto, 190 Elizabeth Street, R. Fraser Elliott Building, 3-805, Toronto, ON M5G 2C4 Canada; 2 Temerty Faculty of Medicine, University of Toronto, 6 Queen’s Park Crescent West, Third Floor, Toronto, ON M5S 3H2 Canada; 3 Department of Medicine, University of Calgary, Foothills Medical Centre – North Tower, 9Th Floor, 1403 – 29th Street NW, Calgary, AB T2N 2T9 Canada; 4 Hotchkiss Brain Institute, University of Calgary, 3330 Hospital Dr NW, Calgary, AB T2N 4N1 Canada; 5 O’Brien Institute of Public Health, University of Calgary, 3280 Hospital Dr NW, Calgary, AB T2N 4Z6 Canada; 6 Knowledge Translation Program, Li Ka Shing Knowledge Institute, St. Michael’s Hospital, 209 Victoria Street, East Building, Toronto, ON M5B 1W8 Canada; 7 St. Michael’s Hospital, 36 Queen St East, Toronto, ON M5B 1W8 Canada

**Keywords:** Diagnostic accuracy, Electronic health literacy tools, Electronic health literacy, E-health literacy, Electronic information literacy, Digital literacy, Computer literacy, Older adults, Systematic review

## Abstract

**Background:**

In Canada, virtual health care rapidly expanded during the COVID-19 pandemic. There is substantial variability between older adults in terms of digital literacy skills, which precludes equitable participation of some older adults in virtual care. Little is known about how to measure older adults’ electronic health (eHealth) literacy, which could help healthcare providers to support older adults in accessing virtual care. Our study objective was to examine the diagnostic accuracy of eHealth literacy tools in older adults.

**Methods:**

We completed a systematic review examining the validity of eHealth literacy tools compared to a reference standard or another tool. We searched MEDLINE, EMBASE, CENTRAL/CDSR, PsycINFO and grey literature for articles published from inception until January 13, 2021. We included studies where the mean population age was at least 60 years old. Two reviewers independently completed article screening, data abstraction, and risk of bias assessment using the Quality Assessment for Diagnostic Accuracy Studies-2 tool. We implemented the PROGRESS-Plus framework to describe the reporting of social determinants of health.

**Results:**

We identified 14,940 citations and included two studies. Included studies described three methods for assessing eHealth literacy: computer simulation, eHealth Literacy Scale (eHEALS), and Transactional Model of eHealth Literacy (TMeHL). eHEALS correlated moderately with participants’ computer simulation performance (*r* = 0.34) and TMeHL correlated moderately to highly with eHEALS (*r* = 0.47–0.66). Using the PROGRESS-Plus framework, we identified shortcomings in the reporting of study participants’ social determinants of health, including social capital and time-dependent relationships.

**Conclusions:**

We found two tools to support clinicians in identifying older adults’ eHealth literacy. However, given the shortcomings highlighted in the validation of eHealth literacy tools in older adults, future primary research describing the diagnostic accuracy of tools for measuring eHealth literacy in this population and how social determinants of health impact the assessment of eHealth literacy is needed to strengthen tool implementation in clinical practice.

**Protocol registration:**

We registered our systematic review of the literature a priori with PROSPERO (CRD42021238365).

**Supplementary Information:**

The online version contains supplementary material available at 10.1186/s12877-023-03899-x.

## Background

Virtual care is used 4.6 times more often by older adults than before the COVID-19 pandemic [1]. Virtual care has rapidly expanded in most care sectors in Canada during the pandemic [2]. Despite this rise in virtual care use, older adults participate less in videoconference-based assessments than their younger counterparts and they predominantly use telephone as opposed to videoconference-based assessments [3]. Videoconference-based virtual care is uniquely complex in comparison to telephone-based care, as it requires the patient to be able to access and navigate webpages and webcam technology. Patients must possess a level of electronic health (eHealth) literacy in order to successfully navigate online healthcare videoconferencing platforms to communicate with their physician [4]. Not only are telephone-based assessments suboptimal because clinicians cannot see patients, but there is greater diagnostic uncertainty associated with telephone as opposed to videoconference-based cognitive assessments [4, 5]. Further, inexperience with technology created unreadiness among older adults towards accessing healthcare via videoconferencing [6]. Reduced use of videoconferencing and barriers associated with its use among older adults suggest a digital divide and uncertainty about how rapidly evolving virtual care practices are addressing older patients’ needs and concerns [6–9].

To tackle the digital divide, we must be able to assess eHealth literacy. eHealth involves health information and services provided via the Internet and other technologies, including virtual care, forums, electronic health records, and smartphone applications to facilitate healthcare decision-making [10–12]. eHealth literacy consists of more than computer literacy because it also incorporates traditional medical and information literacy [13]. Higher eHealth literacy is associated with improved cognitive health and low eHealth literacy is associated with poor medication adherence and increased risk of cardiac events in older adults [14, 15].

Increased uptake of virtual care, specifically the need to use videoconference-based assessments due to our greater certainty in their diagnostic accuracy compared to telephone-based assessments, indicates an urgent need for evaluation of eHealth literacy skills [16]. Clinicians and patients are concerned about the accuracy, effectiveness of virtual assessments and online health interventions, and older adults’ eHealth literacy skills [17–25]. An accurate method for assessing eHealth literacy would enable providers to predict if patients may have difficulty accessing virtual care and provide appropriate support to help them access virtual care. Given these concerns and the diagnostic uncertainty associated with how to assess older adults’ eHealth literacy skills, we completed a systematic review examining the diagnostic accuracy of eHealth literacy tools in older adults.

## Methods

We reported our systematic review as per the Preferred Reporting Items for Systematic reviews and Meta-Analysis of Diagnostic Test Accuracy Studies (PRISMA-DTA) and Synthesis without meta-analysis (SWiM) guidance [26, 27]. This systematic review protocol was registered with PROSPERO (CRD42021238365) [28].

### Data sources and search strategy

We searched MEDLINE, EMBASE, CENTRAL/CDSR, and PsycINFO for citations in any language. Our search was conducted from inception until January 13^th^, 2021. We used controlled vocabulary and keywords related to clusters of terms for eHealth Literacy and Older Adults (details in Supplementary File 1). Grey literature was identified using the Canadian Agency for Drugs and Technologies in Health (CADTH) Grey Matters Guide, following the Grey Literature Checklist and study authors’ content knowledge on July 26^th^, 2021 (Supplementary File 2) [29]. We searched references of included studies. Our search strategy was created and reviewed by authors (YQH, ZG, JAW) and a librarian experienced in developing systematic review literature searches (JM).

### eHealth literacy reference standard

As of now, there is no agreed-upon reference standard for measuring eHealth literacy [16, 30]. We considered computer simulation or direct observation of eHealth-related tasks as the reference standard, and we made an a priori decision to include studies comparing two electronic health literacy assessments in older adults. We included articles that described eHealth literacy as either a general eHealth literacy tool or a disease-specific eHealth literacy tool. For articles that met our inclusion criteria and did not report diagnostic accuracy outcomes (e.g., sensitivity, specificity), we emailed authors to see if these data were available.

### Study selection

All articles with data related to diagnostic accuracy comparing one eHealth literacy tool to a reference standard or another tool, where the mean population age was at least 60 years old, were eligible for inclusion. Upon completing our systematic review, we realized that our participant age inclusion criteria (enrolling subjects of 60 and older with the mean age of 65 and older – initially selected based on the definition from Centers for Disease Control and Prevention) was too restrictive, and we revised our criteria to include all studies where the mean population age was 60 years of age or older [31]. We chose the mean population age of 60 years of age or older as it is the threshold defined by United Nations for “older persons” [32]. All abstracts were reviewed independently, in duplicate by four authors (YQH, LL, JAW, ZG), and any abstract included by either author was reviewed at the full-text stage. Two authors (YQH, LL) independently reviewed all full-text articles; disagreement was resolved by discussion and a third author (JAW), if needed. We calculated the Cohen’s kappa coefficient (κ) using SAS University Edition to determine inter-reviewer agreement on article selection [33].

### Data abstraction and quality assessment

Two reviewers independently (YQH, LL) abstracted data from each included full-text article and appraised the risk of bias using the Quality Assessment for Diagnostic Accuracy Studies-2 (QUADAS-2) tool [34]. Discrepancies were resolved within reviewer pairs and adjudicated by a third reviewer (JAW). We abstracted aggregate-level data from included studies such as name of the first author, study design, year of publication, country where the study was conducted, sample size, study setting (e.g., geriatric medicine clinic, general practitioner clinic), names of tools compared, participant’s primary language, demographic characteristics and experience in Internet use, number of items on each tool, reported cut-offs on tools, and reference standard used for measuring eHealth literacy. We abstracted data as per the PROGRESS-Plus framework, which is suggested by the Cochrane Handbook to assess the inclusion of social determinants of health in included studies [35, 36].

### Synthesis

We could not complete a meta-analysis of diagnostic accuracy outcomes because there were too few included studies.

## Results

**Fig. 1:**
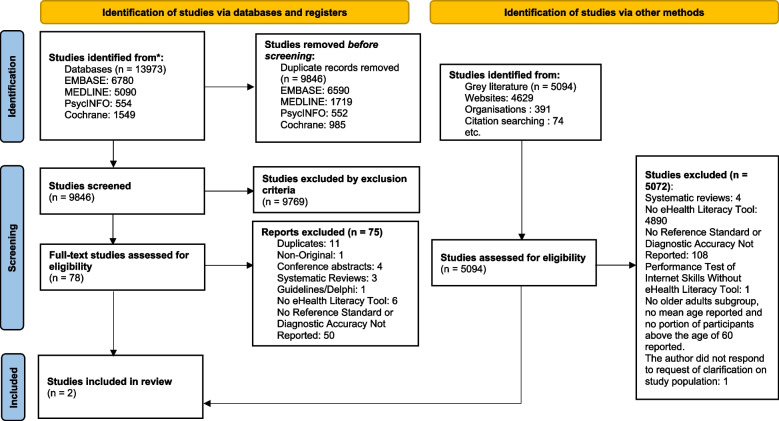
Preferred Reporting Items for Systematic Reviews and Meta-Analysis (PRISMA) flow diagram

We screened 14,940 titles and abstracts and 99 full-text articles, which resulted in two included studies (365 participants) (Fig. 1). Agreement between reviewers who completed full-text article screening was excellent (κ = 0.89; 95% confidence interval 0.82—0.97). The corresponding author of one included article, Neter et al., provided further data specific to the group of adults who were at least 60 years old [37]. Of the 50 excluded studies, the most frequently used tool was the eHealth literacy scale (eHEALS) (*n* = 45) [13, 38]. Most articles were excluded because they did not include a comparator group.

### Included articles

#### Neter et al.

This study enrolled 82 community-dwelling older adults living in Israel; 83% were of Jewish ethnicity (Table 1) [37]. The mean population age was 66.9 years old, and the study population was predominantly female (60%), well-educated (72% graduated from high school) and earned above-average income (53%) [37]. Further ethnicity data, religion, occupation, social support and personal characteristics such as frailty or disability and time-dependent relationships such as hospitalization or respite care were not reported [37]. Fifty-one of 82 individuals who underwent eHEALS testing and computer simulation were aged 60 years or older as per subgroup data provided by the study author [37]. The primary study outcome was the correlation between eHEALS (a self-reporting tool) score and computer simulation performance [37]. eHEALS is an 8-item self-reporting tool that assesses an individual’s perceived eHealth skills using a 5-point Likert scale to answer each question; response options range from “strongly agree” to “strongly disagree” (maximum score of 40) [38]. The cut-off value for eHEALS in this study was defined as the mean score of participants (Table 2) [37]. The computer simulation consisted of 15 tasks to be completed within an allotted time frame, which reflected participants’ operational, formal, information and strategic skills (total time of 108 min) [37]. Each participant received a rating on each task, which ranged from “not completed” to “completed independently” and the amount of assistance required was noted [37]. eHEALS scores (“perceived eHealth literacy”) correlated moderately to computer simulation results (“performed eHealth literacy”), with a correlation coefficient *r* of 0.34 (*p* < 0.01) [37].

**Table 1:** Study characteristics and diagnostic accuracy outcomes

First Author, Year of Publication	Population included	Sample Size	Average Age (years)	Female (%)	Education (years)	Income (level)	Language/Ethnicity	Country	Comorbidities	Internet Use Experience (years)
Neter et al., 2017 [37]	Israeli adults of 50-year-old and above	82^a^ 223^b^	66.95	60%	72% of the total sample had grade 12 and above education	53% of the total sample had average income and above	Hebrew, Arabic and Russian (% not reported) 83% of the total sample was Jewish, no other ethnicity data was given	Israel	43% of the total sample had chronic conditions	12.16
Paige et al., 2019 [39]	Individuals from an US university-based research registry, of 40-year-old and above	283	64.34	56.5%	95% had grade 12 and above education	51.6% had average income and above	Language used was not reported 90.1% white non-hispanic, 3.18% African American and 3.18% Multi-Racial	United States	50.5% had COPD diagnosis with a moderate degree of respiratory symptom severity, mean of 2.3 chronic illnesses	NR

*Abbreviations: NR* Not Reported

^a^Participants completed the survey and the computer simulation

^b^Participants completed the survey only

**Table 2:** eHealth literacy tools

First Author, Year of Publication	eHealth Literacy Tool						Reference Standard/Comparison					
	Test	Number of Items	Rater	Answer Type	Cut-off	Average Test Score	Test	Number of Items	Rater	Answer Type	Cut-off	Average Test Score
Neter et al., 2017 [37]	eHEALS	8	Self	Likert scale (1–5)	Mean score of eHEALS	2.96 out of 5	Computer skills simulation	15 simulation tasks	Observer	Evaluation of performance in 5 categories (0–35)	Median score of 28	22.65 out of 35
Paige et al., 2019 [39]	TMeHL	18	Self	Likert scale (1–5)	NR	NR	eHEALS	8	Self	Likert scale (1–5)	NR	NR

*Abbreviations: eHEALS* eHealth Literacy Scale, *TMeHL* Transactional Model of eHealth Literacy, *NR* Not Reported

#### Risk of bias assessment of Neter et al.

There was high risk of bias for patient selection as study recruitment was completed by telephone and 90% of surveyed participants withdrew from the study before participating in the computer simulation component; additional participants were recruited by snowball sampling (Table 3) [37]. There was no reporting of standardization of the telephone interviews to mitigate bias from the administrator such as a formalized script or training of the interviewers. The risk of bias from reference test (computer simulation) administration was unclear because the training and inter-rater reliability of the reference standard assessors were not reported. Index (eHEALS) and reference (computer simulation) tests had low applicability concerns [37].

**Table 3:** Quality assessment of diagnostic accuracy studies (QUADAS-2)

Study Identification	Risk of Bias				Applicability Concerns		
Author, Year	Patient Selection	Index Test	Reference Standard	Flow and Timing	Patient Selection	Index Test	Reference Standard
Neter et al., 2017 [37]	High	High	Unclear	Unclear	High	Low	Low
Paige et al., 2019 [39]	High	Unclear	High	Unclear	High	Unclear	High

#### Paige et al.

This study included 283 community-dwelling older adults from a university-based research registry in the United States (Table 1) [39]. The mean population age was 64.3 years, and participants were predominantly White (90.1%), female (56.5%), well-educated (95% had an education level of high school and higher) and earned more than $50,000 annually (51.6%) [39]. Authors did not report social determinants of health such as religion, social capital and personal characteristics or time-dependent relationships [39]. The study’s primary outcome was the correlation between TMeHL and eHEALS [39].

TMeHL is an 18-item self-reporting tool with four to five items under functional, communicative, critical and translational literacy [39]. TMeHL uses a 5-point Likert scale for each item (maximum score of 90) [39]. There was no cut-off value proposed to identify sufficient eHealth literacy for TMeHL (Table 2) [39]. The internal validity of TMeHL was determined via dimensionality and item analysis. The external validity of TMeHL was assessed through a comparison of scores to eHEALS (no added cut-off value by the study author), other online health information-seeking styles, and a health literacy tool (the All Aspects of Health Literacy Scale [AAHLS]) [39–42]. TMeHL had a moderate-to-high positive correlation with eHEALS on all four components of eHealth literacy: functional (*r* = 0.47; *p* < 0.01), communicative (*r* = 0.63; *p* < 0.01), critical (*r* = 0.66; *p* < 0.01), and translational (*r* = 0.65; *p* < 0.01) scales [39].

#### Risk of bias assessment of Paige et al.

There was a high risk of bias related to patient selection and applicability because individuals from a university research registry were recruited into the study via an email survey, which would select participants with higher digital literacy (Table 3) [39]. It was unclear if the index and reference standards were interpreted independently [39]. Unclear risk of bias from flow and timing primarily reflected a lack of reporting of the time between administration of index and reference standards [39]. The eHEALS as a reference standard has a high risk of bias because it has not been externally validated [43]. It was unclear if index test results were interpreted without knowledge of the reference standard [39].

## Discussion

We found two tools that will support clinicians in measuring older adults’ eHealth literacy [37, 39]. However, both studies had components of their risk of bias assessments at unclear or high risk of bias and only one study assessed the external validity of eHEALS. Neter et al. found a moderate correlation between eHEALS and computer simulation [37]. TMeHL had a moderate-to-high correlation compared to eHEALS, but authors did not compare TMeHL to a reference standard [39]. Further, important social determinants of health such as social capital were not reported, which limits our understanding of how health equity factors influence the diagnostic accuracy of tools measuring eHealth literacy in older adults in older adults. Although we highlight limitations in our understanding of how eHEALS and TMeHL can be used to assess older adults’ eHealth literacy, as listed above, our systematic review is important because it is the first systematic review reporting on the diagnostic accuracy of tools for measuring eHealth literacy in older adults and our findings further a timely conversation about how we can equitably support older adults in accessing videoconference-based care, mobile health tools, and other digital health solutions.eHEALS was the most widely used eHealth literacy tool, as per our systematic review. Studies have validated eHEALS’ internal consistency, but not its external validity [38, 44–46]. For example, one study evaluating the internal validity of eHEALS found a Cronbach’s coefficient of 0.94 when re-tested eight weeks later in a group of educated older adults with high internet use; construct validity was evaluated by relating eHEALS score to individual Internet use, which was gathered via surveys [47]. Studies that solely validate eHEALS through the construct validity concept of Internet use are insufficient to represent the exhaustiveness of eHealth literacy such as the six spheres of the Lily Model [13]. This is further supported by a recent systematic review of studies assessing eHealth literacy tools’ ability to measure the competence areas of eHealth literacy against the European Commission’s Digital Competence (DigComp) framework [48, 49]. eHEALS only covered one out of five criteria of the DigComp framework. We did not identify any eHealth literacy tools within the two included studies that were evaluated based on all three subtypes of validity either (that is, content, construct, and criterion), which are imperative to ensure methodological quality of a tool’s measurement properties [50]. Further, Lee et al. showed that eHEALS had inconsistent low-quality evidence for relevance and insufficient very low-quality evidence for comprehensiveness [51]. On the other hand, eHEALS had moderate to high-quality evidence in structural validity, internal consistency, and measurement invariance [51]. Further research will be needed to fill these gaps in our understanding of the validity of eHEALS as a tool for measuring eHealth literacy in older adults.

**Fig. 2:**
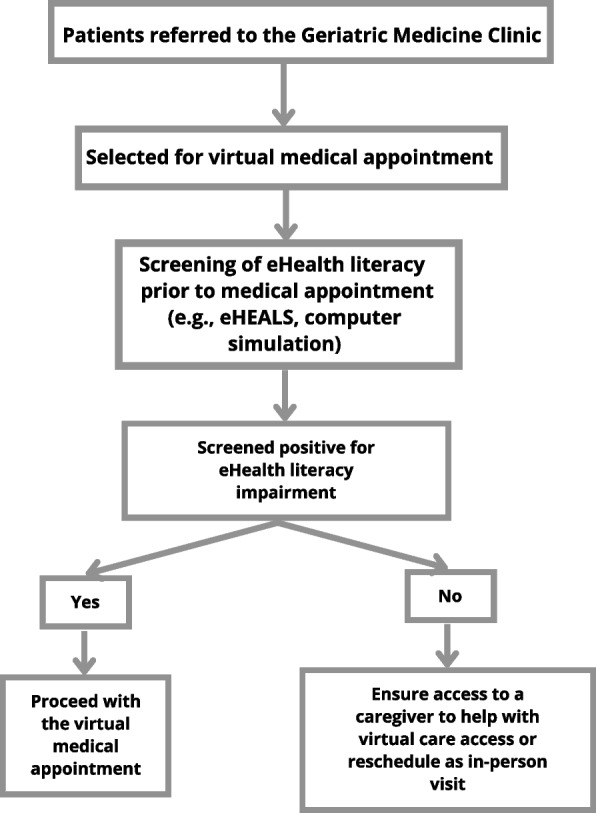
Flow diagram illustrating implementation of eHealth literacy tools included in our review in a geriatric medicine clinic

Not being able to assess older adults’ eHealth literacy represents a critical knowledge gap and barrier to the sustainability of digital health solutions, especially as virtual care is integrated into routine healthcare delivery [7]. Further, there is a burgeoning interest in interventions to improve older adults’ eHealth literacy, especially in terms of technology use and internet and mobile applications; however, how can these interventions be developed and tested if there is no agreed-upon reference standard for assessing eHealth literacy and the diagnostic accuracy of tools for assessing eHealth literacy has not been compared to this reference standard [43, 52–54]? Griebel et al. summarized multiple definitions of eHealth literacy and underlined the importance of agreeing on an updated definition of eHealth literacy [55, 56]. Despite global efforts to develop eHealth literacy tools, there is no eHealth literacy tool of reference, even in adults of other age groups (< 65 years of age) [51, 57]. Evidence suggests that tools may be excessively restrictive in scope (disease-specific or not accounting for the rise of social media and mobile web) [51, 57]. The implementation of tools for assessing older adults’ eHealth literacy will be strengthened by further research to standardize the definition of eHealth literacy and understand a tool’s external validity and the influence of social determinants of health (Fig. 2).

Our systematic review has limitations. First, we could be missing relevant articles; however, we were inclusive in our database search and grey literature search. Second, there were too few studies to complete a meta-analysis of diagnostic accuracy estimates. Third, included studies had small sample sizes with limited recruitment strategies. Recruited participants were predominantly Israeli and White. As illustrated by our equity analyses following the PROGRESS-Plus framework, the applicability of these two eHealth literacy tools is limited as there wasn’t diverse representation within the small sample size [35]. Moreover, both studies’ participants were not patients requiring medical attention or intervention; they were on a national telephone registry or part of a university-based research registry. Thus, these findings may not be applicable to the clinical setting. Lastly, there was no description of personal characteristics such as cognitive impairment or frailty, among other factors, for participants in included studies; hence, our findings may not be generalizable to a population of older adults attending a geriatric medicine clinic. To overcome this limitation, future validation studies will need to include more diverse populations of older adults seeking medical care and describe the potential impact of geriatric syndromes on the assessment of eHealth literacy.

## Conclusions

In conclusion, we completed the first systematic review on the diagnostic accuracy of eHealth literacy tools in older adults. We identified two eHealth literacy tools that were compared to a reference standard or another tool (that is, eHEALs and TMeHL); however, study limitations such as incomplete reporting of diagnostic accuracy measures (e.g., lack of sensitivity or specificity for studied tools) and unclear to high risk of bias across multiple components of each study’s risk of bias assessment preclude us from recommending one tool over another. Future research describing the sensitivity and specificity of tools for measuring eHealth literacy in older adults and how social determinants of health impact the diagnostic accuracy of eHealth literacy tools would strengthen tool implementation in clinical practice.

## Supplementary Information

**Additional file 1: Supplementary file 1.** Search strategy.

**Additional file 2: Supplementary file 2.** Grey literature databases searched.

## Data Availability

The data can be found in Table 1. The studies included in our systematic review were published in peer-reviewed manuscripts and available on MEDLINE.

## Abbreviations

eHealth: Electronic health
eHEALS: EHealth Literacy Scale
TMeHL: Transactional Model of eHealth Literacy
PRISMA-DTA: Preferred Reporting Items for Systematic reviews and Meta-Analysis of Diagnostic Test Accuracy Studies
SWiM: Synthesis without meta-analysis guidance
CADTH: Canadian Agency for Drugs and Technologies in Health
κ: Cohen’s kappa coefficient
QUADAS-2: Quality Assessment for Diagnostic Accuracy Studies-2
AAHLS: All Aspects of Health Literacy Scale
r: Correlation coefficient
DigComp: European Commission’s Digital Competence
