# Supplementary material for: Diagnostic accuracy of eHealth literacy measurement tools in older adults: a systematic review
Source: BMC Geriatr. 2023 Mar 29;23:181. doi: 10.1186/s12877-023-03899-x (PMC10049781; doi:10.1186/s12877-023-03899-x)
Supplement: Supplementary file 1 — Additional file 1: Supplementary file 1. Search strategy. [file 12877_2023_3899_MOESM1_ESM.docx]

**Additional File 1: Supplementary File 1.** **Search strategy**

Database(s): Ovid MEDLINE: Epub Ahead of Print, In-Process & Other Non-Indexed Citations, Ovid MEDLINE® Daily and Ovid MEDLINE® 1946-Present

| **#** | **Searches** | **Results** |
| --- | --- | --- |
| 1 | exp Health Literacy/ | 6021 |
| 2 | exp Information Literacy/ | 6088 |
| 3 | exp Computer Literacy/ | 1675 |
| 4 | (eHealth or digital health or electronic health or e-literacy or electronic health literacy or e-health literacy or digital health literacy or online health literacy or health information literacy).tw,kf,ab. | 23762 |
| 5 | 1 or 2 or 3 or 4 | 31317 |
| 6 | limit 5 to "all aged (65 and over)" | 5251 |
| 7 | (geriatric* or elder* or older* or ageing or aging or senior* or older adult* or retired or retiree* or elder* or pensioner* or nursing home* or older people or older patient* or gerontology or Sexagenarian* or septuagenarian* or octogenarian or nonagenarian* or centenarian* or sixties or seventies or eighties or nineties).tw,kf. | 1896390 |
| 8 | exp Aged/ or geriatrics/ or aging/ or Aged, 80 and over/ | 3294394 |
| 9 | 7 or 8 | 4531451 |
| 10 | 6 and 9 | 5251 |
| 11 | exp "Sensitivity and Specificity"/ | 586855 |
| 12 | exp "Predictive Value of Tests"/ | 203823 |
| 13 | exp ROC Curve/ | 58601 |
| 14 | exp "Surveys and Questionnaires"/ | 1040853 |
| 15 | exp Mass Screening/ | 128666 |
| 16 | exp Validation Study/ | 101854 |
| 17 | (valid* or screen* or tool* or questionnaire* or instrument* or test* or psychometric* or survey* or neuropsych* or inventory* or diagnos* or sensitivit* or specificit*).kf,tw,ab. | 8052919 |
| 18 | 11 or 12 or 13 or 14 or 15 or 16 or 17 | 8573294 |
| 19 | 10 and 18 | 3899 |
