# Supplementary material for: Diagnostic accuracy of eHealth literacy measurement tools in older adults: a systematic review
Source: BMC Geriatr. 2023 Mar 29;23:181. doi: 10.1186/s12877-023-03899-x (PMC10049781; doi:10.1186/s12877-023-03899-x)
Supplement: Supplementary file 2 — Additional file 2: Supplementary file 2. Grey literature databases searched. [file 12877_2023_3899_MOESM2_ESM.docx]

**Additional File 2: Supplementary File 2. Grey literature databases searched**

1. TRIP database
2. Agency for Healthcare Research and Quality
3. Government of Canada
4. GreyNet International
5. SIGLE (System for Information on Grey Literature in Europe)
6. Center for Research Libraries Foreign Dissertation
7. DART-Europe E-theses Portal
8. Electronic Theses Online Service (ETHOS) | British Library
9. Open access dissertations
10. Thesis Canada Portal
11. NICE (National Institute for Health and Care Excellence)
12. SIGN (Scottish Intercollegiate Guidelines Network)
13. Google Scholar
